# Heterogeneities in the latent functions of employment: New findings from a large-scale German survey

**DOI:** 10.3389/fpsyg.2022.909558

**Published:** 2022-08-17

**Authors:** Sebastian Bähr, Bernad Batinic, Matthias Collischon

**Affiliations:** ^1^Department Panel Study “Labor Market and Social Security,” Institute for Employment Research, Nuremberg, Germany; ^2^Institute of Education and Psychology, Johannes Kepler University Linz, Linz, Austria

**Keywords:** latent functions of employment, Jahoda, labor market, employment, unemployment

## Abstract

Building on social psychologist Marie Jahoda’s pioneering work, the psychological literature has shown that work fulfills both manifest functions (e.g., monetary returns) and latent functions (e.g., social contact). This article uses data from the German panel study “Labor market and social security” (PASS), which contains information on latent and manifest factors (from a shortened latent and manifest benefits, or LaMB, scale), as well as a large array of other variables for over 9,000 respondents. This probability-sampled data allowed for detailed analyses that have not been previously possible. We investigate differences in these factors by labor market status, among those employed, and among those unemployed. We identify considerable variation between status groups, suggesting that employment, overall, is important and that longer periods of unemployment lead to a gradual decay of the latent and manifest factors. Furthermore, regression analyses show that the LaMB measures account for approximately 70% of the partial correlations between unemployment and various well-being measures.

## Introduction

Marie Jahoda’s latent deprivation model (1981, 1982) provided a pioneering theoretical framework for understanding the psychological effects of unemployment. The roots of this theory stretch back to the Marienthal study, in which the sociopsychological effects of unemployment were studied; away from the laboratory, a whole set of empirical socio-psychological research methods had been newly developed (Jahoda et al., 1933). Jahoda differentiates between the obvious, manifest function of work, such as income, from the five so-called latent functions that work fulfills for individuals. Work provides the following: (1) *collective purpose*, the opportunity to contribute to a higher collective purpose, thus, linking an individual to goals and purposes that transcend their own; (2) *status and identity*, as work defines certain aspects of status and recognition; (3) *social contacts*, in the sense of regularly shared experiences and contacts with people outside the nuclear family, thus, enlarging social horizons; (4) required *activity*; and (5) a *time structure* for the waking day, the week, and the year (Jahoda, 1981, p. 188).

According to Jahoda (1982), these benefits of work, which can only be measured indirectly, satisfy basic human needs. She argues that unemployment is related to reduced access to these latent functions of work and that unemployed persons “do not enjoy their “leisure”; they become disheartened, lose their self-respect and their sense of time, and felt on-the-scrap heap (Jahoda, 1981, p. 189). Consequently, deprivation of these latent functions harms the mental health of unemployed individuals.

Jahoda’s model served as a stimulus for several other theoretical approaches to explaining the relationship between mental health and unemployment. Following Jahoda, Warr (1987), for example, developed the so-called “vitamin model,” a theory of the effect of work and unemployment on people’s mental health. The model lacks the essential distinction between manifest and latent functioning. According to the model, nine contextual factors or “vitamins,” which are very similar to the functions of work according to Jahoda, provide for the working person, as well as for the unemployed person, thus, protecting their mental health. According to Warr, a low supply of vitamins is generally detrimental to mental health. Depending on the vitamin, a high supply leads to an ever-increasing positive effect, no effect, or a negative effect. The theoretical approaches of Jahoda and Warr assume that external factors affect a person’s mental health. Fryer’s agency restriction model Fryer (1986) stays in contrast with this. It focuses on the financial impoverishment of unemployed people and postulates that this deprives people of their freedom of action, which ultimately has a negative impact on their mental health.

Jahoda’s latent function model also inspired several empirical studies in the interdisciplinary field of research on unemployment. Although Jahoda focused on mental health, later studies broadened the scope by also investigating the relationship of unemployment with well-being and life satisfaction (Hetschko et al., 2021). Based on identity theory, and in direct connection with Jahoda’s latent function status and identity, Hetschko et al. (2014) investigated the transition to the retirement of employed and unemployed people with regard to life satisfaction. They found that long-term unemployed people report a substantial increase in their life satisfaction when they retire. Using German panel data, Kunze and Suppa (2017) found negative effects of unemployment on social participation, i.e., unemployed individuals experienced negative effects with regard to social activities, as well as withdrawal of individuals into private life, which highlights the importance of the latent function of “social contacts” among unemployed people. A major difference between these exemplarily listed studies and Jahoda’s research model is that the latent functions of work are not examined simultaneously.

Besides replicating existing results, this article contributes to the literature in three ways. First, we provide novel evidence on the variation of the latent and manifest benefits of work (LaMB) by employment status groups, using a representative sample covering over 9,000 respondents. Second, we investigate variation in the specific functions within employment and unemployment, along the lines of pay, working hours, and unemployment durations.

The experience of unemployment is stressful (Fryer and Payne, 1986; Jahoda, 1988). Meta-analyses of longitudinal studies show that unemployment is causally responsible for decreased well-being and mental health. The longer individuals are unemployed, the more negative effects are observed (McKee-Ryan et al., 2005; Paul and Moser, 2009; Krug and Eberl, 2018). The latent deprivation model provides a possible explanation for the harmful effects of unemployment on health. Empirical studies have been able to support the assumption that deprivation regarding the latent functions of work varies according to occupational status and that unemployed individuals report lower levels of health compared to full-time employed individuals, as well as in comparison to persons who are out of the labor force, i.e., students, homemakers, and retirees (Creed and Reynolds, 2001; Paul and Batinic, 2010). Several cross-sectional and longitudinal studies have also found evidence that latent functions are related to well-being and psychological health (cf. Haworth and Paterson, 1995; Wanberg et al., 1997; Creed and Macintyre, 2001; Waters and Moore, 2002; Hoare and Machin, 2010; Paul and Batinic, 2010; Selenko et al., 2011; Muller and Waters, 2012; Huffman et al., 2015). Furthermore, Zechmann and Paul (2019) found through a 6-wave study (*N* = 1,061 participants at t_1_) that re-employment predicted gains in the latent functions of work, which reduced distress. An interesting approach is taken by Houssemand and colleagues in their work (Pignault and Houssemand, 2017; Houssemand et al., 2021) by pointing to a possible dynamic development of the relationship between latent functions and health and emphasizing the importance of unemployment normalization. Unemployment normalization has a cognitive and an emotional dimension and represents a coping strategy for the unemployed that cushions the possible negative effects of unemployment on health. While the mediating role of the latent functions of work in the relationship between unemployment and health outcomes seems well supported, we do not know the extent to which these latent functions of work decrease with increasing unemployment duration.

Given the increasing number of non-standard work arrangements (Smith and Halpin, 2019), the question about the different levels, to which the latent functions of work are fulfilled, becomes even more relevant because the traditional contrast of employment and unemployment no longer corresponds to the increasingly heterogeneous labor market. The notion of non-standard work arrangements is an umbrella term for work arrangements that deviate from permanent full-time employment (Tanimoto et al., 2021) in different ways. The most common non-standard work arrangement is part-time work. To date, it has been unclear whether the number of working hours matters for the satisfaction of the latent functions of work. Similarly, the wage level could also function as an indicator of integration into the labor market and job quality. If LaMB levels vary by job characteristics, we would expect to find variation in wages.

If LaMB satisfaction is less dependent on having a job or not but varies finer-grained, then we would also expect heterogeneity in unemployment. The literature has repeatedly shown that the duration of unemployment is indicative of the psychological reaction to it (see e.g., Paul et al., 2018), but there is no clear pattern of the effect. In the context of fulfilling the latent functions of work, the influence of unemployment duration has not yet been sufficiently investigated.

Differentiating among the analyses on the impact of the latent functions of work reveals that their effects on well-being and psychological health vary considerably (see also Creed and Bartrum, 2006; Hoare and Machin, 2006). For example, Batinic et al. (2010) conducted two studies to investigate whether latent functions of work mediate the relationship between occupational level and well-being. In study 1, this assumption was supported for the latent functions of time structure and status, while in study 2, it is for time structure and social contact but not for status (Batinic et al., 2010). In a study conducted by Creed and Macintyre (2001), with a sample of 248 unemployed people on the relationship between the manifest and latent functions of work and well-being, the authors concluded that while both manifest and latent benefit contribute significantly to predictions of well-being, the manifest benefits make the largest contribution. They concluded that status was the most important contributor among the latent functions, followed by time structure and collective purpose. Sousa-Ribeiro et al. (2014) found in a sample of 300 Portuguese adults (*n* = 133 unemployed, 91 employed full-time, and 76 unemployed attending a full-time training course), overall, that “all of the latent benefits, except of status, were found to be negatively related to psychological distress and social contact, while collective purpose and activity were positively associated with satisfaction with life” (p. 646). For predicting well-being and quality of life, Trewick and Muller (2014) found that some benefits of employment were important, while others are not (for well-being: time structure, collective purpose, status, and financial income; for quality of life: time structure, social contact, status, and financial income).

In addition to the rare findings on the relative impact of the latent functions on health outcomes, even less is known about the role of potential relative effects between the manifest and the latent functions of work. The fact that individual latent functions, such as status, are closely related to income is problematic. It is reasonable to assume that both constructs share common variance components. Selenko et al. (2020) took an interesting approach to this issue by relating the latent functions of work to unpaid volunteer work. Through two longitudinal studies conducted in Germany and the United Kingdom, the authors found evidence that volunteering increased the latent benefits (see also Yang and Matz, 2020). These results indicate that the latent functions also exist independently of the manifest function of work. This finding contradicts the assumption by Jahoda (1982) that in modern societies, one can obtain all five latent functions to a sufficient extent only through employment and that, therefore, any job is preferable than being unemployed. Concerning the latent functions of work, however, Selenko et al. (2020) also found differences between the two countries regarding the importance of the individual latent functions. In the German sample, collective purpose and social contacts increased in magnitude over time, while in the United Kingdom sample, the same increase was found for time structure and activity.

Against this backdrop, Jahoda only differentiated between paid work and unemployment when investigating the latent functions. She makes no differentiated claims about the extent to which different jobs satisfy the latent functions to different levels (Hoare and Machin, 2006). With this in mind, Batinic et al. (2010) applied the latent functions theory of work to white- and blue-collar workers and examined these two groups regarding their well-being and access to the latent functions of work. The results revealed that “the two occupational-level groups differed significantly regarding psychological well-being and access to latent benefits of work. People in higher-level occupations had significantly better access to latent benefits and reported significantly better psychological well-being than respondents in low-level occupations” (Batinic et al., 2010, p. 84). This finding suggests that different occupations can be classified based on the extent to which they fulfill the latent functions and that latent functions could also be used when designing work. However, the corresponding research is largely lacking.

To summarize, there are various open questions concerning the work- and job-related determinants of the latent functions of work. Studies of latent functions often rely on small samples that are ambiguously representative (one of the few exceptions here is the study by Paul and Batinic, 2010). From a theoretical point of view, it remains unclear whether the latent functions depend solely on the distinction between working and not working or whether there is variation even within employment constellations in terms of non-standard work arrangements, working hours (Creed and Machin, 2002), and wages. Furthermore, the relationships discovered are often unstable, and replications of studies lead to different results concerning the impact of the latent functions, e.g., for health outcomes.

The objectives of this study are to examine the following four central questions:

(a) What is the relationship between work’s latent and manifest functions and employment status/weekly working hours?
(b) What is the relationship between wages and the satisfaction of the manifest and the latent functions of work?
(c) What effect^1^ does the duration of unemployment have on the manifest and latent functions of work?
(d) What is the contribution of the five latent functions and the manifest function of work to explanations of the variation in well-being and health?

To this end, we use newly available data from a German panel study that covers a large number of respondents and provides a rich set of variables that can be used in the analysis, thus, enabling us to investigate the questions above (for more details, see the next section). Therefore, in contrast to previous studies, we are less constrained by power issues and concerns of sample selection. Thus, these rich and powerful data provide us with the opportunity to take a detailed look at variation in the latent and manifest benefits that have not been possible in previous studies.

This article is structured as follows: the “Methods” Section presents the data and relevant variables used in the analyses, and then discusses the empirical strategy. The “Results” Section presents the results of the analyses. The “Discussion and implications” Section concludes and discusses the limitations of the study, as well as potential avenues for future research.

## Methods

### Data

We analyzed data from 2020, stemming from the household panel study “Labor market and social security” (PASS), a survey conducted by the IAB (Institute for Employment Research of the Federal Employment Agency, Germany) and designed to facilitate research on the labor market and poverty in Germany (Trappmann et al., 2019). The data are particularly well suited for this study. First, PASS includes rich information on individuals’ employment situations, socio-economic characteristics, and household contexts, allowing for unprecedentedly detailed subgroup analyses of specific employment groups. Second, PASS consists of two large random samples: one of the German populations and one of welfare benefit recipients. With 10,210 individuals from 7,547 households, PASS provides large-scale data that is representative of Germany, particularly for welfare benefit recipients, a group consisting of both long-term unemployed individuals and marginalized employees, thus, especially relevant to the study of the latent functions of work.

The welfare benefits in Germany are the basic source of income support for households of working-age people with insufficient income (Federal Employment Agency, 2021). Household members receive payments irrespective of their labor market status. Thus, welfare recipients need not be unemployed but can be members of the working poor or be temporarily outside the labor force (e.g., due to childcare or illness). As of May 2020, in the middle of PASS’s annual field survey period, 8.1% of working-age people received this form of support, with 16% currently employed in some form, 30% unemployed, and 58% inactive^2^ (Federal Employment Agency, 2020). In our main analysis, we do not use survey weights to account for the sampling scheme, but we do control for potential factors related to selectivity as recommended by Solon et al. (2015). However, as a robustness check, we apply German population weights (Trappmann, 2013) in our analyses to compensate for the over-representation of welfare benefit recipients in the PASS data – the results are virtually unchanged although the standard errors slightly increase (see Supplementary Appendix Figure A1 of the online Supplementary Appendix).

Furthermore, we would like to point out that the data were collected when the COVID-19 pandemic hit, and consequently, the fieldwork switched from mixed mode to telephone interviews only. Thus, we also present the main descriptive results by survey timing – before and during the lockdown in Germany – to ensure that our results are unbiased by either the crisis or the change in survey mode from mostly in-person interviews to telephone interviews.

#### Analysis sample

From an initial sample of 10,210 participants from 2020, we ended up with 9,303 participants in our analysis sample. We drop 493 observations by conditioning the non-missing LaMB factor information and lose an additional 414 observations due to missing values in the control variables and health outcomes, as described in the next section.

### Measures

#### The latent and manifest benefits module

Latent and manifest benefits of work were measured with the German version of the shortened Latent and Manifest Benefits of Work Scale (LaMB) from Kovacs et al. (2017). Based on two larger empirical studies (*N* = 1054; *N* = 677), the scale’s authors conclude that their version of LaMB represents an economical, i.e., cost-effective, instrument with satisfactory psychometric properties. The scale is based on the instrument of Muller et al. (2005), which has been used in numerous publications on the latent functions of work (Muller and Waters, 2012). The LaMB scale consists of 18 items, where the five latent benefits (collective purpose, social contact, status, activity, and time structure) and financial strain, a (negatively poled) manifest benefit, are each measured with three items. The respondents scored each item on a seven-point Likert scale (“completely disagree” to “completely agree”; see Supplementary Appendix Table A1 for the exact wording).

We constructed the six LaMB factors from standardized additive indices^3^ using Stata’s alpha command with case-wise deletion. By construction, the resulting factors have means of zero and standard deviations (SD) approaching one. Table 1 displays the pairwise correlations between the six factors and Cronbach’s alpha reliability coefficients. Except for factor 5, which is the time structure, all factors display sufficient internal consistency. To adhere to the LaMB scale, we, nevertheless, use factor 5 in our analyses (Supplementary Appendix Table A2 shows an expanded table containing correlations with all other model variables).

**TABLE 1:** Correlations between latent and manifest functions.

	LAMB Factor 1: Collective purpose	LAMB Factor 2: Social contact	LAMB Factor 3: Status	LAMB Factor 4: Activity	LAMB Factor 5: Time structure	LAMB Factor 6: Financial strain	Alpha scores
LAMB Factor 1: Collective purpose	1						0.826
LAMB Factor 2: Social contact	0.504***	1					0.678
LAMB Factor 3: Status	0.508***	0.492***	1				0.765
LAMB Factor 4: Activity	0.148***	0.106***	0.113***	1			0.789
LAMB Factor 5: Time structure	0.418***	0.337***	0.404***	0.096***	1		0.582
LAMB Factor 6: Financial strain	−0.407***	−0.407***	−0.263***	−0.184***	−0.306***	1	0.887

*p < 0.05, **p < 0.01, ***p < 0.001.

#### Measures of well-being

We analyze the extent to which the latent and manifest functions of work helped explain well-being and health. While Jahoda (1981, 1982) addressed psychological well-being in the context of latent deprivation, in our analysis, we broaden the focus to address general health-related well-being, as well as psychological well-being. Both of these constructs are multidimensional with no single standard operationalization, which is why we use several suitable instruments that are available in the PASS data (for details, see: Trappmann et al., 2019; Müller et al., 2020).

##### Health satisfaction

The *domain-specific* and *general life satisfaction* instruments in PASS are taken from the German Socioeconomic Panel Study (SOEP) (Schimmack et al., 2008). For our analysis, we focus on respondents’ satisfaction with their health. Respondents could score the item on an eleven-point scale ranging from “completely dissatisfied” to “completely satisfied” (see Supplementary Appendix Table A3 for the exact wording of the item).

##### Subjective general health and subjective mental health

The subjective assessment of general and mental health, which are both based on the SF-12 short survey (Ware et al., 1995; Nübling et al., 2006), is a tested objective and a comprehensive generic health measure (Ziebarth, 2010). Both items asked respondents to rate their health in the 4 weeks preceding the interview on a five-point rating scale (see Supplementary Appendix Table A3 for the exact wording and answer labels).

##### Good general health and good mental health

For each of the two items above, we also constructed dichotomous variables that take on a value of one if respondents rate their general health as “good” or “very good” or report “no” or “few” mental problems. We use these additional outcomes to assess the overall robustness of the results and ensure that the potential effects in the five-point scales are not only driven by changes within already good or bad health categories, but they also reflect jumps from bad or average to good health (see Supplementary Appendix Table A6 and Supplementary Appendix Figure A2). Table 2 displays descriptive statistics for all instruments.

**TABLE 2:** Sample descriptive statistics.

	Range	Median	Mean	Std. Dev.
Health satisfaction	0–10	7	6.81	2.16
Subjective general health*	1–5	3	3.30	1.05
Good general health*	0/1	–	0.45	–
Subjective mental health*	1–5	4	3.65	1.27
Good mental health*	0/1	–	0.60	–
Employment status: Reg unemployed	0/1	–	0.17	–
Employment status: Inactive	0/1	–	0.36	–
Employment status: Minijob	0/1	–	0.09	–
Employment status: Part-time	0/1	–	0.13	–
Employment status: Full time	0/1	–	0.24	–
Age	15–95	50	48.25	16.25
Female	0/1	–	0.51	–
Years of education	7–21	12	12.58	3.04
Partner in household	0/1	–	0.52	–
Children under 15 in household	0/1	–	0.25	–
CAPI interview mode (vs. CATI)	0/1	–	0.21	0.41
Hourly wages (in euros, employed only)	1–837	16	20.23	64.34
Actual working hours (employed only)	1–95	39	35.97	11.52
Unemployment duration (in months; unemployed only)	1–603	32	46.11	50.35
*N* = 9,303				

*Results for the additional health measures are shown in the online Supplementary Appendix.

#### Regressors

##### Employment status

Our primary focus is on analyzing how groups defined by employment status differ in obtaining the latent and manifest benefits of work. The PASS data provide fine-grained employment information, which allows for detailed subgroup analyses. We distinguish full-time work from part-time work (we define full-time work as employees with more than 35 contractual working hours per week) and marginal employment, called “Minijobs” in Germany. Minijobs are subject to limited social security, with contributions paid by employers only for gross earnings up to €450 per month.

To address subgroup heterogeneity, we control for age (in years), gender, years of education, the presence of a partner or children in the household, as well as the interview mode: either a computer-assisted personal interview (CAPI) or a computer-assisted telephone interview (CATI).

Table 2 shows the overall summary statistics for our sample. The sample is balanced by gender, but as indicated by employment status, more than 50% of the respondents are inactive (out of the labor force) or registered as unemployed – a result of the sampling procedure described previously. We accounted for these imbalances by controlling the employment status in our analysis. In addition to information on the institutional employment setting (full-time, part-time, or Minijob), we also applied detailed information on *actual working hours* (including regular overtime) and the resulting gross *hourly wages*.^4^

However, the sampling procedure also allowed us to focus on heterogeneities in the factors among those who are unemployed. Here, we analyzed different *unemployment durations (in months)*. The median time out of work for unemployed respondents was 18 months, with some outliers with very long but plausible durations. The large sample size for long-term unemployed individuals is a unique feature of the PASS data that enables unprecedented investigations of non-immediate changes in the LaMB factors.

#### Analytical strategy

Our empirical analysis relied on ordinary least square (OLS) regressions to account for the confounding effects of covariates, such as age, that could be correlated with the latent factors that we are interested in, as well as the main regressor of interest, such as employment status. In the first step, we estimate the following equation:


(1) factori=β0+β1EMPSTAT+′iβ2X+′iϵi

where *factor*_*i*_ is the latent or manifest function of interest. Thus, we estimate the regression six times – one estimation for each factor. *EMPSTAT*_*i*_ is individual *i*’s employment status, and *X_i_* is a set of covariates that account for potential confounding effects. *ϵ*_*i*_ is an idiosyncratic error term. In the baseline model, the covariates include age, age^2^, gender, and education, whether a partner is living in the respondent’s household, whether there are children under the age of 15 in the household, and interview mode (as the interview mode switched from telephone and in-person interviews to telephone interviews only due to the COVID-19 pandemic). We use these control variables to control for sample composition and interview-induced effects (for the survey mode). All variables were considered in passing the test as suggested by Bernerth and Aguinis (2016), as they are likely correlated with the outcomes and can be measured reliably. We abstained from controlling for further factors, because any labor-market-related characteristics could be outcomes of the latent factors as well and could be considered ‘‘bad controls’’ in this sense^5^. In this specification, we are solely interested in variation in the individual factors by employment status.

In the next step, we have dived deeper into the variation in the factors among those employed and those unemployed by, again, estimating separate models for employed and unemployed individuals. The former estimation for employed individuals controls for occupation (with the 4-digit-ISCO), monthly gross pay and actual working hours categories. It could be the case that certain jobs are only available with a given number of working hours or a certain wage level. Thus, we need to account for occupation to isolate the effect of pay and working hours. With these estimations, we investigated variation in the latent factors across the range of working hours and pay while holding occupation constant. In the estimation for unemployed individuals, we analyzed variation in the latent factors by unemployment duration to investigate whether the latent functions deteriorate as time spent out of employment increases.

Finally, we investigate whether latent functions can explain the correlation between unemployment and various measures of well-being. In these estimations, we first estimate the following model:


(2) yi=β0+β1EMPSTAT+′iβ2X+′iϵi

where *y*_*i*_ is the outcome of interest, e.g., life satisfaction; we are interested in β_1_, which is an estimate of the mean differences in *y*_*i*_ by employment status. We are especially interested in the differences between those who have full-time employment and those who are unemployed to assess the negative effects of unemployment on well-being. In the next step, we add the latent factors to the model:


(3) yi=β0+β1EMPSTAT+′iβ2Xi′+δfactori′+ϵi

δ is the coefficient indicating the relation between the latent functions and well-being. Furthermore, we compare the estimates of β_1_from estimations (3) and (2) to assess whether differences in the latent functions can explain the effects of unemployment. To this end, we also conducted a formal mediation analysis (Preacher et al., 2007; Preacher and Hayes, 2008) to assess the share of the effect of unemployment on various health outcomes that is mediated by the loss of latent and manifest functions. In all estimations, we used heteroscedasticity-robust standard errors.

We also wanted to stress that because only the cross-sectional data are available at the moment, all findings presented in the next section are descriptive in nature and cannot be interpreted causally. Nevertheless, we think that analyzing the differences in latent functions by employment status and investigating their partial correlations with well-being measures have merits in its own right and provide avenues for further future analysis.

## Results

### Latent and manifest functions by employment status

**FIGURE 1:**
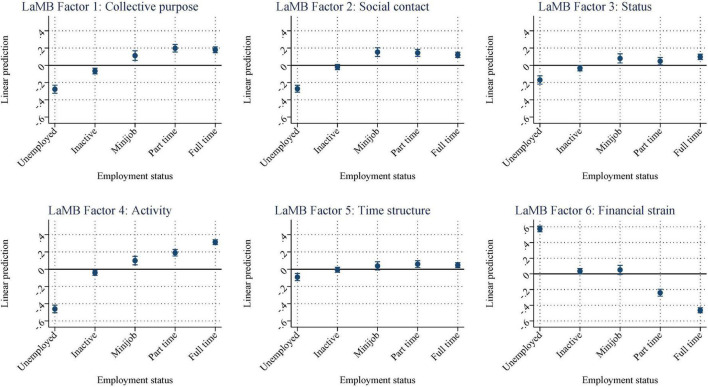
Standardized LAMB scores by employment status. Supplementary Appendix Table A6 shows the corresponding regression results.

We began investigating the differences in the latent functions by estimating Equation (1). Figure 1 presents the predicted mean values from (1) by employment type (Supplementary Appendix Table A4 shows the unweighted, unaltered means of the factors by subgroup; Supplementary Appendix Table A6 shows the corresponding regression coefficients used to create the figure). Please note that we allowed the scales of the six panels to vary; thus, highlighting the variance between employment status groups within each panel. For comparisons of the magnitudes of the coefficients between panels, see Supplementary Appendix Tables A4, A5. Furthermore, the horizontal line at zero represents the sample mean and does not indicate whether the size of any given coefficient differs significantly from zero.

There is a substantial variation in the LaMB factors. Overall, the *collective purpose* (LaMB factor 1) seems to be provided by employment, whereas inactivity or unemployment is associated with a lower degree of collective purpose. Individuals in Minijobs and part-time employment score highest on the *social contact* factor (LaMB factor 2), thereby suggesting that there might be a trade-off between working time, contacts through working, and personal contacts. However, both inactive and unemployed individuals show below-average scores for this factor. LaMB factor 3, *status*, is similar among employed individuals, but again, unemployed and inactive respondents scored below average.

There seems to be a linear relationship between attachment to the labor market and *activity* (LaMB factor 4): unemployed respondents scored lowest, while full-time employees scored highest on this scale. However, *time structure* (LaMB factor 5) shows only slight variation by employment type. Nevertheless, unemployed individuals are also disadvantaged in terms of this factor.

The most significant difference in magnitude is found in the sixth factor, which is the manifest factor: *financial strain* (LaMB factor 6). As expected, unemployed individuals report the highest levels of financial strain, while such strain is lowest for full-time employees. Inactive individuals are an exception to this pattern. However, their inactivity could imply that these individuals are not actively looking for work, indicating that they possess sufficient financial resources within the household to forego labor income. Overall, the results indicate stark differences in the LaMB factors between employed and unemployed individuals and within different types of employment. Supplementary Appendix Figures A1, A2 further show that our results are robust using the survey’s design weights, changes in the interview mode, and pandemic-induced effects. Next, we use our rich data to dive deeper into the heterogeneities in the LaMB factors within subgroups.

### Latent and manifest functions within employment and unemployment

Next, we investigated the variation in the factors among those who are employed. To this end, we regressed the latent factors on indicators for different working hours and gross hourly pay, occupation, and the set of covariates from the previous estimations. Our sample includes employed individuals only. We are now interested in whether the factors differ by pay or more fine-grained measures of working time or whether having a job has a binary effect on the latent factors.

**FIGURE 2:**
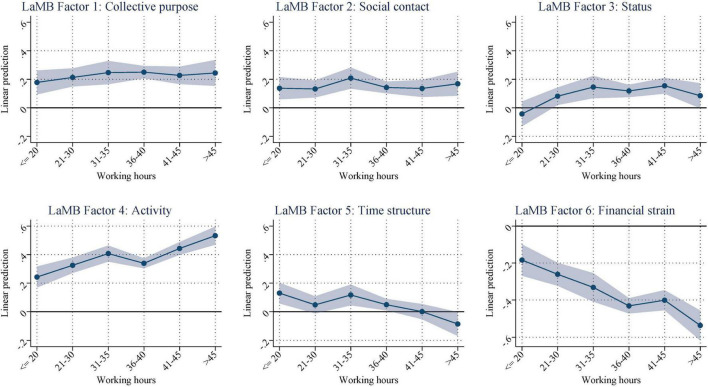
Conditional on working: predicted scores by categories for hours worked (net of the effects of an hourly wage, welfare benefit receipt, age, age squared, gender, education, partner in HH, child (under 15) in HH, 4-digit-ISCO, and interview mode). Supplementary Appendix Table A7 shows the corresponding regression results.

Thus, Figure 2 displays the predicted means from the regressions by working time while holding hourly pay constant (Supplementary Appendix Table A7 shows the corresponding coefficient estimates). With this specification, we analyzed the extent of temporal integration into the labor market without interference from the effects of earning a higher income. As shown in the figure, *collective purpose*, *social contact*, and *status* seem to barely differ across the working hours’ distribution. *Time structure* decreases slightly from approximately 35 h of work. In contrast, there is a clear upward trend in *activity* across working time, while *financial strain* decreases.

**FIGURE 3:**
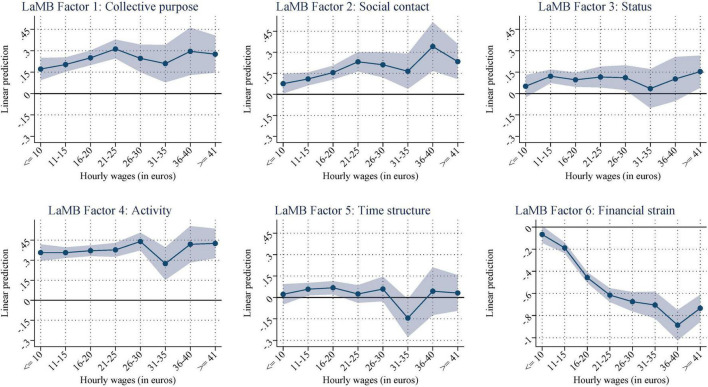
Conditional on working: predicted scores by hourly wage categories (net of the effects of working hours, welfare benefit receipt, age, age squared, gender, education, partner in HH, child (under 15) in HH, 4-digit-ISCO, and interview mode). Supplementary Appendix Table A7 shows the corresponding regression results.

Next, in Figure 3, we investigated the variation across the hourly pay distribution while holding working time constant (again, Supplementary Appendix Table A7 shows the corresponding coefficient estimates). Here, we focused on the differences in the status and prestige of employment positions, independent of total income.

*Collective purpose* and *social contact* both initially increase with rising hourly wages but level off once wages exceeded 20 euros, suggesting that these dimensions benefited from pay only up to a certain degree. This trend seems to be weaker for s*tatus*. *Activity* and *time structure* seem to be unaffected by the different wage levels, which is not surprising given that we also hold working time constant. Furthermore, *financial strain* decreases with higher pay, which is not surprising but also shows that this factor is closely related to pay.

**FIGURE 4:**
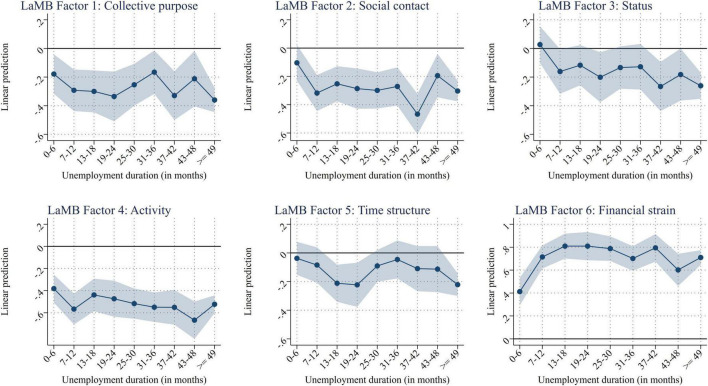
Conditional on unemployment: predicted scores by monthly unemployment duration categories (net of age, age squared, gender, education, partner in HH, child (under 15) in HH, and interview mode). Supplementary Appendix Table A8 shows the corresponding regression results.

Next, we investigated the variation in the factors among unemployed individuals (excluding inactive individuals). For this group, the factors might not deteriorate instantaneously when entering unemployment but may gradually deteriorate over time. Thus, we investigate the effect of unemployment duration on the functions in Figure 4 (Supplementary Appendix Table A4 shows the unweighted, unaltered means of the factors by unemployment benefits group^6^; Supplementary Appendix Table A8 shows the corresponding coefficient estimates).

As shown in the figure, most factors remain remarkably constant over time but are significantly below the sample average. Several worsen over time; *social contact*, for example, decreases while *financial strain increases*. Again, *status* seems to be determined outside the labor market, indicating a lack of perceived stigmatization (e.g., Gurr and Jungbauer-Gans, 2017). However, there seem to be adaptive processes at play to some degree. For example, *collective purpose*, and *time structure* increase again after 25 months of unemployment. To the best of our knowledge, there is no institutional explanation for this increase. We thus assume that these findings potentially reflect adaptation (Georgellis et al., 2008; Witte et al., 2010; Paul et al., 2018). However, we do not want to overinterpret these findings, as the increases are never statistically significant. Furthermore, there could be time-variant selection in and out of unemployment for those who suffer most from unemployment (i.e., those whose latent functions deteriorate the most in the initial phase of unemployment, see, Suppa, 2021). Additionally, the status may especially be prone to endogeneity in the sense that the meaning of the status measures shifts if unemployed individuals over time replace employed with unemployed social contacts in their social network (also, see text footnote 7). Thus, these findings need to be interpreted with caution.

Overall, these results not only show that employment status seemed to have a binary effect on the latent functions but also that there is a substantial variation among those employed and those unemployed. For employees, some of the latent and manifest functions differ by pay and working time, even within jobs (because we control for the 4-digit-ISCO classification of occupations). For unemployed individuals, financial strain increases over time. These results show that investigating the latent and manifest functions within employment statuses provides some nuances that have, thus far, not been shown. Nevertheless, employment, overall, is beneficial for most factors, as displayed in Figure 1.

### Latent functions as predictors of well-being

To investigate whether the latent and manifest functions reported in the survey are predictive of other important outcomes, we investigated whether they could explain the effects of unemployment on various well-being measures. To this end, we regressed each well-being measure of interest on employment status and the set of covariates that we used previously, then compare the coefficient estimates for employment status to a model, in which we additionally controlled for the latent and manifest functions. In all cases, full-time regular employment is the reference category. Thus, all coefficient estimates for the other status categories show differences relative to this category.

**FIGURE 5:**
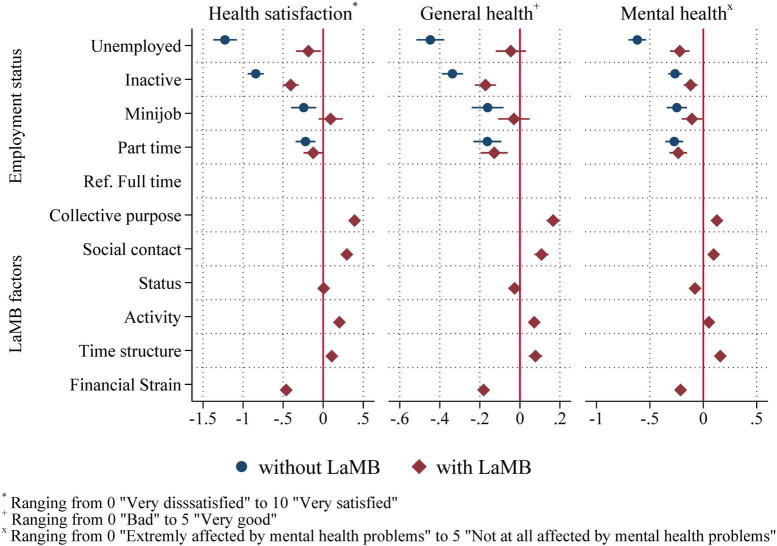
Coefficient plot from regressions of well-being measures on employment status and LaMB factors (net of the effects of age, age squared, gender, education, partner in HH, child (under 15) in HH, and interview mode). 95% CI. Both outcome scales range from 0 “completely dissatisfied” to 10 “completely satisfied.” The results correspond to columns 1 to 4 in Supplementary Appendix Table A9.

In our analyses, we focus on the aforementioned measures of health satisfaction, general health, and mental health. Figure 5 shows the results of the estimations (Supplementary Appendix Table A9 shows the coefficient estimates). Note that the confidence intervals for the employment status coefficients are wider than those for the LaMB coefficients because the former variables are discrete, and the latter are continuous. Since all estimations produce very similar results, we focus mainly on the results for health satisfaction as an example. Note that the outcomes in the regression results are not standardized. Thus, coefficients can be compared for the same outcome, but not in terms of magnitude between different outcomes.

The left panel of Figure 5 shows the results for health satisfaction. The blue circles in the left panel of Figure 5 (Supplementary Appendix Table A9, column 1) show the estimates obtained without controlling for the LaMB factors. As the figure shows, net of controls, registered unemployed individuals exhibit, on average, a 1.1-point lower score on the health satisfaction scale. The magnitude of this coefficient is substantial: the −1.1-point disadvantage experienced by unemployed individuals relative to individuals with full-time employment corresponds to an approximately 15% lower level of health satisfaction (compared to the mean for full-time employees of 7.375 on the eleven-point scale). The red diamonds (column 2) show the estimates from the same specification but with the latent and manifest function measures added.

All factors also correlate significantly with health satisfaction, except for *status*, whereas the manifest function, *financial strain*, exerts the strongest effect in terms of coefficient magnitude. This finding is consistent with previous research that found that while both manifest and latent benefits contribute significantly to predictions of well-being, the manifest benefits make the largest contribution (Creed and Macintyre, 2001). Furthermore, the share of the variation in health satisfaction explained by the model (measured with *R*^2^) increases from 14.4% to 25.1% (displayed in Supplementary Appendix Table A9) after adding the latent and manifest functions to the regression. Concerning the coefficients on the latent and manifest functions, the results show that most factors are significantly correlated with health satisfaction in the direction that one would expect; higher scores on the latent functions correlate positively with health satisfaction, while greater financial strain is associated with reduced health satisfaction. The zero effect that we estimated regarding *status* fits the previous findings and could result from the notion that status is always relative, and unemployment shifts the reference frame but does not decrease status overall.

Furthermore, we conducted a formal mediation analysis for each outcome, as proposed by Preacher and Hayes (2008). The lower part of Supplementary Appendix Table A9 shows the indirect effect of unemployment on the respective outcome through the LaMB-variables and the share of the total effect that is explained by the factors. The LaMB measures explain approximately 75% of the partial correlation between unemployment and health satisfaction. These findings show that the latent and manifest functions are also highly predictive of a relevant outcome.

Furthermore, we repeated this exercise with additional health outcomes, specifically subjective general health, as well as mental health, on a scale from 1 (poor) to 5 (excellent). The results are displayed in the middle and right panels of Figure 5 (and the corresponding columns in Supplementary Appendix Table A9). The results for both outcomes largely mirror the results for health satisfaction: the negative coefficient on unemployment drastically decreases in magnitude when latent functions are added to the model and is even rendered statistically insignificant in the case of general health. The mediation analyses reveal that the LaMB-variables explain 68% of the estimated effect of unemployment on mental health and around 85% of the effect of unemployment on general health (lower parts of Supplementary Appendix Table A9). Thus, the LaMB-variables seem to play an important mediating role from unemployment to various health measures. Additionally, Supplementary Appendix Figure A3 shows the results when using the binary indicators for good general and mental health (points 4 and 5 on the self-assessment scales, respectively) as outcomes (the corresponding columns in Supplementary Appendix Table A10 show the coefficient estimates)^7^. These results closely mirror the main results presented previously: accounting for latent and manifest factors drastically decreases the partial correlation between unemployment and the respective well-being measure. Furthermore, Supplementary Appendix Figure A4 displays the results obtained without the inclusion of control variables. Again, the picture remains clear: the LaMB variables explain a large share of the impact of unemployment on the respective well-being outcomes. Overall, the results thus also show that latent and manifest functions are essential determinants of the correlation between unemployment and health and that these functions are predictive of health, regardless of the scale used.

## Discussion and implications

This article identifies variation in Jahoda’s latent and manifest functions of work along various dimensions in a large sample of the German population. We find that the latent and manifest functions show substantial variation by labor market participation status and that unemployed individual are generally more deprived in terms of these functions. Investigating the heterogeneities within the employed and unemployed groups shows that conditional on employment, working for more hours is associated with higher values for activity and lower values for financial strain, while the other functions do not vary much beyond employed individuals, exhibiting generally high levels. Higher pay, with holding working hours constant, is associated with higher collective purpose, social contact, and lower financial strain. Within the unemployed group, nearly all dimensions deteriorate over time but with some adaptive processes at work in between. Using linear regression analyses, we further found that latent functions can explain a large share of the negative correlation between unemployment and health and well-being outcomes, regardless of the instrument used to assess well-being.

To the best of our knowledge, this study is the first to analyze Jahoda’s latent and manifest functions with a large population survey. Overall, the findings highlight the benefits of using extensive and detailed data for such analyses by providing insights into heterogeneities that cannot easily be analyzed with smaller datasets.

### Discussion

This study contributes to understanding the role of latent and manifest benefits of employment by investigating these concepts within a large German population survey. Thus, we overcame issues arising from small sample sizes and selective samples and are, thus, able to provide detailed evidence on the variation of Jahoda’s functions within employment and unemployment. Furthermore, we confirm that these functions are instrumental in explaining the reduced mental health of the unemployed and expand this finding to other established health outcomes.

Our study provides further evidence for the assumption that unemployed individuals report lower access to the latent functions and the manifest function of work than individuals employed full-time. This difference, however, varies across the latent functions and is particularly small for time structure. This result is noteworthy because several studies have highlighted the importance of time structure and psychological distress for predicting well-being (Creed and Macintyre, 2001; Hoare and Machin, 2006; Batinic et al., 2010). However, a descriptive analysis of the data also shows that the differences among the occupational status groups (Minijobs, part-time/full-time employees) are mostly evident in the manifest function. Among the latent functions, differences are limited to activity. This result may suggest that (a lack of) access to the latent functions of work is a problem for unemployed people but that the extent, to which one works plays only a minor role in determining deprivation levels. Thus, it only matters that one has worked, but not how much one actually works.

Occupational status is closely related to the number of hours worked and is sometimes even defined in terms of the number of hours worked per week. If we look at the number of hours a person works and relate this value to his or her access to the manifest and latent functions of work, we obtain a picture characterized by wide differentiation. Although the number of hours worked per week is still linearly related to the manifest function of work, this is only observable for the latent function in the case of activity. On the other hand, higher weekly working time leads to reductions in the latent function time structure. The three latent functions of collective purpose, social contact, and status are satisfied relatively equally across weekly working times of up to 45 h.

In summary, we conclude that it is essential to simply have work to satisfy the latent functions of work and that the extent of employment is less important. More work leads to a slight increase in the satisfaction of individual latent functions and, thus, has a slightly positive effect. A high workload (45 or more working hours per week), on the other hand, reduces access to the latent function time structure and is thus counterproductive.

Examining the hourly wage distribution (keeping working hours constant) also reveals differentiation in the latent functions. That is, collective purpose and social contacts increase up to an hourly wage of 20 euros but then remain constant. A possible explanation could be that the focus of the constituting questions (see Supplementary Appendix Table A1 for the translated wording) is not limited to coworker networks but focus on the respondent’s more general social status within his or her personal network. Network homophily^8^ (McPherson et al., 2001) could help explain these relatively stable effects. The other latent functions are affected little by the different wage levels. Moreover, financial strain decreases with higher wages, but this is to be expected.

The duration of unemployment is related to well-being and health (Paul and Moser, 2009). Nevertheless, many studies on the latent functions of work simply compare employed people with unemployed people and do not make distinctions concerning the length of unemployment. The reason for this is usually less theoretical and is primarily the result of the small sample sizes used in those studies. Such small samples do not allow for a fine-grained differentiation of the unemployed group concerning the length of unemployment. However, this is precisely what we were able to do in this study. The results of our study show that some latent functions decrease and that financial strain increases with an increasing unemployment duration.

In accordance with Jahoda’s assumption that latent deprivation sets in immediately after a job loss, we found deprivation regarding *collective purpose*, *social contact*, *activity*, and *financial strain* from the first 6 months of unemployment onward. While individual reactions to unemployment may vary, overall, we found a slight variation in the LaMB factors throughout unemployment. For most unemployed, unemployment insurance benefits run out after 12 months and are replaced by the lower welfare benefits. While we cannot see direct differences in deprivation between the two schemes (see Supplementary Appendix Table A5), the deprivation regarding *social contact*, *activity*, and *financial strain* worsens significantly at 12 months (see also Supplementary Appendix Table A8). Given we use cross-sectional data, our results could also be caused by selection in and out of unemployment and, thus, should be interpreted with caution. More detailed subgroup analyses considering the literature on unemployment adaptation [see, e.g., the meta-analysis of Paul and Moser (2009)] and future longitudinal analyses are promising avenues for future research.

Regarding the analysis of LaMB’s role in explaining the correlation between unemployment and various well-being measures, we can show that being unemployed is strongly and negatively correlated with well-being. This effect is largely explained (approximately 70%) by variation in the latent and manifest functions. In line with previous findings (Creed and Macintyre, 2001), manifest and latent functions are significantly correlated with well-being. The results of these analyses further underline our previous finding: work matters, as employed individuals reported higher levels of well-being than unemployed individuals. Our findings also show that the concept of latent and manifest functions still has important implications today, as such functions explain a large share of this correlation.

### Limitations and implications

Our study also has several limitations that could be addressed in future research. First and foremost, the information on the latent and manifest functions that we used is currently only available cross-sectionally. Thus, it is difficult to draw causal conclusions, as we still lack panel information that is important to identify causal effects. For example, we cannot exclude the argument that latent functions do not deteriorate within unemployment but that the selection of individuals changes over time and only those with specific characteristics remain unemployed for long periods. In addition, various psychological scales were used in this study, which has been used many times in the past and is well-documented but, ultimately, only the indirectly measured subjective components. However, these scales measure only indirectly subjective states, and objective measures would certainly be preferable here. Furthermore, regarding our analyses of the connection between well-being and the latent and manifest functions, it could be the case that decreases in a particular function leading to unemployment and not vice versa; i.e., there could be reverse causality. Fortunately, more data are currently being collected that will allow for panel analysis, thus, at least, resolving several selection issues and enabling researchers to account for individual fixed effects in the analysis. Nevertheless, truly claiming causality requires exogenous variation, e.g., through institutional reforms.

Data collection of our study took place between February and September 2020. Thus, we need to address the impact and the changes induced by COVID-19 not only on work and life in general but also on the survey fieldwork we might have on our findings. In response to rising COVID-19 case numbers, the German government imposed a nationwide lockdown on March 23. Due to Germany’s liberal use of furlough schemes, there were few actual layoffs (Aiyar and Dao, 2021). However, these measures could have influenced our results. Since the first weeks of PASS’s field period fall before the lockdown, we can use this variation to assess its impact on our results.

Supplementary Appendix Figure A2 in the Supplementary Appendix reports the effects of being interviewed before the lockdown relative to being interviewed during the remainder of the field period. We find a stable pattern between employment groups both before and during the pandemic. While there may have been a shift in levels or even notable changes for individual groups, the fundamental pattern in the fulfillment of the LaMB factors between employment status groups remains. Thus, we conclude that our results should reflect a stable pattern of LaMB fulfillment and not short-term effects due to unique circumstances.

In light of all the changes that have occurred in society, the labor market, and social security institutions in the 40 years since Marie Jahoda first formulated the latent functions of work, it is fascinating to find her fundamental principles confirmed so clearly. Having any work increases collective purpose, social contact, status, and activity above those of the unemployed or inactive population. Whether one has a full-time job or only a marginal Minijob does not seem to matter. This finding contributes to the ongoing debate about whether marginal employment acts as a stepping stone into the labor market or a poverty trap (Booth et al., 2002; Lietzmann et al., 2017; Boschman et al., 2021). While Minijobs provide latent functions similar to those of other forms of employment, they fail to mitigate financial strain. Therefore, the LaMB scale enables us to paint a more complete picture.

To analyze the heterogeneities within the employed group, we focused on differences in terms of wages and hours worked. While these subgroups are undoubtedly important, this is hardly an exhaustive analysis. The PASS data provide several opportunities for more in-depth approaches to heterogeneous employment groups, such as those defined by industry, firm, occupation, task, and interactions. However, such detailed analyses are beyond the scope of this article but should provide a fruitful avenue for future research.

Jahoda’s claim that the detrimental effect of unemployment on well-being is mitigated to a large degree by latent deprivation was central to her model. We could reproduce this finding with our unique dataset despite its cross-sectional nature. The large sample of long-term unemployed respondents allowed us to identify the changes in LaMB functions over the course of time spent on employment. While most studies can only distinguish between unemployment durations of more and less than 6 months, we can identify unemployment durations of up to 50 months. Our results suggest that latent deprivation does not start immediately but grows continuously over time. Indeed, in the first 12 months, we do not find any substantial changes in the latent functions, which is in line with previous findings (Frasquilho et al., 2016). This threshold coincides with a change in the institutional setting of the unemployment benefit system in Germany. Substantial changes occur in the second year of unemployment, which could also hint at the exhaustion of savings. After this period, we find increased satisfaction of the functions, likely attributable to adaptation to or coping with prolonged unemployment. In addition to institutional conditions that change over time, intrapsychic processes may also be responsible for this dynamic course. In this sense, Houssemand and colleagues highlight in a series of empirical studies the particular importance of unemployment normalization, which is a coping strategy for the unemployed over time and has a cognitive and an emotional dimension (Pignault and Houssemand, 2017; Houssemand et al., 2021). The extension of Marie Jahoda’s model to include these dynamic and changing impact processes over time is very promising in our view. In this context, the use of reliable and sufficient data is vital for drawing conclusions regarding the long-term effects of unemployment.

## Data availability statement

The PASS data are available free of charge for non-profit research as a scientific use file (SUF) at the research data center of the Federal Employment Agency at the Institute for Employment Research. The form to order the data can be accessed at http://fdz.iab.de/en/FDZ_Data_Access/FDZ_Scientific_Use_Files.aspx.

## Ethics statement

Ethical review and approval were not required for the study on human participants in accordance with the local legislation and institutional requirements. Participation in this study was completely voluntary. Written informed consent was obtained from all participants for their participation in this study.

## Author contributions

BB designed the version of the LaMB instrument used. SB implemented the instrument in PASS and supervised the data collection. All authors developed the study design and analysis plan. MC and SB prepared the data and conducted the analyses and data visualization. BB contributed the introduction and literature review to the manuscript, SB the data description, and MC the methods and results section. All authors discussed the results, contributed to the conclusion, and reviewed and edited the final manuscript.

## Footnotes

1 We use the term “effect” for better readability. However, “effect” in this case does not refer to causal effects, but to partial correlations net of control variables.

2 Inactive individuals for example include students, pensioners, homemakers, and other individuals that are neither registered unemployed, nor employed.

3 Confirmatory polychoric factor analysis using the principal factor method and varimax rotation produces factors that have correlations with the alpha scales ranging between 0.88 and 0.99. The results of our analyses do not change when either set of alternative factors is used.

4 Note that some of the values in the data appear large (e.g., 95 working hours last week). However, due to the large number of respondents, rare, but not impossible cases are likely to be included. For transparency’s sake and because they are not impossible, we do not exclude them from the analysis. However, they also do not substantially affect the analysis, because they have a negligible effect on the mean in a sample of this size and because we mostly investigate variation in the latent functions across categories of these variables.

5 We would also like to mention that all results hold even without controlling for the set of covariates; the overall patterns with regards to variation in the latent functions by employment status, as well as in the regression analyses with health outcomes remains the same.

6 There are two institutional regimes for unemployment benefits: the contribution-funded unemployment insurance (called Arbeitslosengeld 1), which is usually paid for up to 12 months and offers a 65% income replacement rate, and welfare benefits (called Arbeitslosengeld 2), a means-tested basic income scheme, which is paid indefinitely to able-bodied working-age individuals.

7 We are aware that the use of five-point scales and dichotomous outcomes in an OLS regression is questionable, as the average effects estimated by OLS may provide only a crude approximation of the true effects if there are non-linearities or may – in the case of binary outcomes – predict outcome values below 0 or above 1. We thus additionally estimated ordered logit models (results not shown) to account for the distribution of the five-point scale responses for the health variables. However, as these estimations fail the Brant test with *p*-values below 0.001, we abstain from interpreting or reporting the results. As a check for the robustness of our overall results for these variables, we also used non-linear models (logit and probit) to recalculate the main results for the dichotomous outcomes for mental and general health. Supplementary Appendix Table A7 shows the average marginal effects from these estimations. The effects differ little from the baseline results in Supplementary Appendix Table A6, highlighting the robustness of our results.

8 Homophily implies, that individuals surround themselves with others of the same employment status, and given prolonged unemployment, replace employed contacts with unemployed ones. Thus, an individual’s status within her personnel network is less dependent on her own employment status.
