# Corrigendum: Heterogeneities in the latent functions of employment: new findings from a large-scale German survey

**DOI:** 10.3389/fpsyg.2023.1243270

**Published:** 2023-07-11

**Authors:** Sebastian Bähr, Bernad Batinic, Matthias Collischon

**Affiliations:** ^1^Department Panel Study “Labor Market and Social Security, ” Institute for Employment Research, Nuremberg, Germany; ^2^Institute of Education and Psychology, Johannes Kepler University Linz, Linz, Austria

**Keywords:** latent functions of employment, Jahoda, labor market, employment, unemployment

In the published article, there was an error. The stated gross earnings limit for Minijobs in Germany is €450 per month, not per week.

A correction has been made to **Methods**, “*Regressors*”, “*Employment status*”, paragraph 1. The corrected paragraph is shown below.

“Our primary focus is on analyzing how groups defined by employment status differ in obtaining the latent and manifest benefits of work. The PASS data provide fine-grained employment information, which allows for detailed subgroup analyses. We distinguish full-time work from part-time work (we define full-time work as employees with more than 35 contractual working hours per week) and marginal employment, called “Minijobs” in Germany. Minijobs are subject to limited social security, with contributions paid by employers only for gross earnings up to €450 per month”.

The authors apologize for this error and state that this does not change the scientific conclusions of the article in any way. The original article has been updated.

