# Supplementary material for: Heterogeneities in the latent functions of employment: New findings from a large-scale German survey
Source: Front Psychol. 2022 Aug 17;13:909558. doi: 10.3389/fpsyg.2022.909558 (PMC9428713; doi:10.3389/fpsyg.2022.909558)
Supplement: Supplementary file 1 [file Data_Sheet_1.pdf]

frontiers

Appendix

to

Heterogeneities in the latent functions of employment: New findings from a large-scale German survey. *Frontiers in Psychology*.

TABLE A1 | LAMB module.

Variable	Label	LAMB factor (polarity)		
PLA0100a	I often feel that I make a meaningful contribution to society.	1	Collective purpose	+
PLA0100b	I often feel a valuable part of society.	1	Collective purpose	+
PLA0100c	I hold a valuable position in society.	1	Collective purpose	+
PLA0100d	I often meet new people.	2	Social contact	+
PLA0100e	I often go out and meet with others.	2	Social contact	+
PLA0100f	I usually have a lot of opportunities to mix with people.	2	Social contact	+
PLA0100g	My friends usually value my company.	3	Status	+
PLA0100h	I am often valued by the people around me.	3	Status	+
PLA0100i	I am usually important to my friends.	3	Status	+
PLA0100j	I often have nothing to do.	4	Activity	-
PLA0100k	I often wish I had more things to do to fill up the time in my days.	4	Activity	-
PLA0100l	There is usually too much spare time in my day.	4	Activity	-
PLA0100m	My days are usually well organized.	5	Time structure	+
PLA0100n	I find it useful to structure my time.	5	Time structure	+
PLA0100o	I have a good balance in my day between responsibilities and free time.	5	Time structure	+
PLA0100p	I often have enough money to buy treats for myself.	6	Financial strain	-
PLA0100q	My income usually allows me to do the things I want.	6	Financial strain	-
PLA0100r	My level of income usually allows me to make plans for the future.	6	Financial strain	-

Note: Items were presented in randomized order.

TABLE A2 Correlations between all model variables

	LAMB Factor 1: Collective purpose	LAMB Factor 2: Social contact	LAMB Factor 3: Status	LAMB Factor 4: Activity	LAMB Factor 5: Time structure	LAMB Factor 6: Financial strain	Health satisfaction (0-10)	Subjective general health (1-5)	Good general health (0/1)	Subjective mental health (1-5)	Good mental health (0/1)	Age (in years)	Female (0/1)	Years of education	Partner in Household (0/1)	Child under 15 in household (0/1)	Hourly wages (in euros)	Actual working hours	Unemployment duration (in months)
LAMB Factor 1: Collective purpose	1.00																		
LAMB Factor 2: Social contact	0.50***	1.00																	
LAMB Factor 3: Status	0.51***	0.49***	1.00																
LAMB Factor 4: Activity	0.15***	0.11***	0.11***	1.00															
LAMB Factor 5: Time structure	0.42***	0.34***	0.40***	0.10***	1.00														
LAMB Factor 6: Financial strain	-0.41***	-0.41***	-0.26***	-0.18***	-0.31***	1.00													
Health satisfaction	0.33***	0.29***	0.21***	0.17***	0.20***	-0.33***	1.00												
Subjective health	0.27***	0.23***	0.16***	0.12***	0.18***	-0.27***	0.66***	1.00											
Good health	0.21***	0.18***	0.14***	0.12***	0.14***	-0.21***	0.52***	0.83***	1.00										
Subjective mental health	0.20***	0.18***	0.11***	0.10***	0.18***	-0.25***	0.34***	0.45***	0.35***	1.00									
Good mental health	0.16***	0.15***	0.09***	0.07***	0.15***	-0.21***	0.28***	0.38***	0.32***	0.87***	1.00								
Age	-0.07***	-0.11***	-0.10***	-0.07***	0.10***	-0.05***	-0.24***	-0.19***	-0.18***	0.07***	0.07***	1.00							
Female	0.05***	0.04***	0.13***	0.12***	0.11***	0.03**	-0.01	-0.07***	-0.06***	-0.14***	-0.13***	0.03**	1.00						
Years of education	0.12***	0.09***	0.09***	0.26***	0.06***	-0.21***	0.14***	0.12***	0.11***	0.04***	0.02*	-0.06***	-0.02*	1.00					
Partner in Household	0.10***	-0.01	-0.02	0.13***	0.08***	-0.21***	0.09***	0.09***	0.07***	0.12***	0.11***	0.16***	-0.01	0.07***	1.00				
Child under 15 in household	0.09***	-0.04***	0.03**	0.12***	0.00	0.03***	0.15***	0.14***	0.13***	0.01	-0.00	-0.35***	0.08***	0.04***	0.26***	1.00			
Hourly wages	0.06***	0.04*	0.00	0.06***	0.04*	-0.18***	0.05**	0.05**	0.03	0.03*	0.03	0.07***	-0.06***	0.20***	0.06***	0.03*	1.00		
Actual working hours	0.01	0.01	0.02	0.13***	-0.08***	-0.16***	0.04**	0.05**	0.06***	0.06***	0.04**	-0.10***	-0.30***	0.08***	-0.00	-0.04*	-0.08***	1.00	
Unemployment duration	-0.18***	-0.18***	-0.15***	-0.19***	-0.08***	0.28***	-0.26***	-0.18***	-0.17***	-0.07***	-0.05***	0.25***	0.00	-0.19***	-0.17***	-0.12***	-0.10***	-0.11***	1.00

Note: * $p < 0.05$, ** $p < 0.01$, *** $p < 0.001$

TABLE A3 | Wording of the health instruments

Variable	Question wording	Answer labels
PA0100	Health satisfaction<br>How satisfied are you today with the following areas of your life? For your assessment, you can use the numbers from "0" to "10". "0" means that you are "very dissatisfied"; "10" means you are "very satisfied". The numbers "1" to "9" allow you to grade your assessment. How satisfied are you with your health?	0 "Very dissatisfied"<br>…<br>10 "Very satisfied"
PG1100	Subjective mental health*<br>How strongly have you been affected by mental problems, such as fear, dejection, or irritability, in the past 4 weeks? Please tell me whether you were affected "not at all", "a little bit", "moderately", "quite a bit" or "extremely"?	1 "Not at all"<br>2 "A little bit"<br>3 "Moderately"<br>4 "Quite a bit"<br>5 "Extremely"
PG1200	Subjective health*<br>How would you describe your state of health in the past 4 weeks in general? Was it. . .	1 "Very good"<br>2 "Good"<br>3 "Satisfactory"<br>4 "Not so good"<br>5 "Bad"

Note: * Both subjective health scores were reverse coded for our analysis.

TABLE A4 | LAMB measures in the data, unconditional – means by employment status

	Reg Unemployed	Inactive	Minijob	Part time	Full time	Total
LAMB Factor 1: Collective purpose	-0.288	-0.100	0.113	0.254	0.208	0.008
	(0.953)	(0.871)	(0.822)	(0.738)	(0.739)	(0.857)
LAMB Factor 2: Social contact	-0.277	-0.029	0.175	0.147	0.128	0.008
	(0.820)	(0.794)	(0.765)	(0.688)	(0.690)	(0.774)
LAMB Factor 3: Status	-0.176	-0.054	0.108	0.116	0.088	-0.004
	(0.971)	(0.863)	(0.779)	(0.723)	(0.694)	(0.827)
LAMB Factor 4: Activity	-0.509	-0.094	0.096	0.314	0.362	0.016
	(0.895)	(0.847)	(0.772)	(0.668)	(0.615)	(0.834)
LAMB Factor 5: Time structure	-0.143	0.034	0.017	0.095	0.008	0.004
	(0.835)	(0.760)	(0.706)	(0.670)	(0.648)	(0.735)
LAMB Factor 6: Financial strain	0.702	-0.064	0.110	-0.194	-0.453	-0.028
	(0.715)	(0.902)	(0.872)	(0.792)	(0.742)	(0.900)

Note: Means (SD); N=9,303.

TABLE A5 | LAMB measures in the data, unconditional – means by unemployment status

	Unemployment insurance (ALG I)	Welfare benefit receipt (ALG II)	Total
LAMB Factor 1: Collective purpose	-0.286	-0.289	-0.289
	(0.971)	(0.950)	(0.953)
LAMB Factor 2: Social contact	-0.236	-0.286	-0.277
	(0.821)	(0.819)	(0.819)
LAMB Factor 3: Status	-0.103	-0.194	-0.178
	(0.984)	(0.968)	(0.971)
LAMB Factor 4: Activity	-0.407	-0.531	-0.510
	(0.910)	(0.891)	(0.896)
LAMB Factor 5: Time structure	-0.0583	-0.159	-0.142
	(0.779)	(0.845)	(0.835)
LAMB Factor 6: Financial strain	0.404	0.762	0.701
	(0.828)	(0.674)	(0.715)

Note: Means (SD), N=1,601.

TABLE A6 | REGRESSION RESULTS CORRESPONDING TO FIGURE 1 (STANDARDIZED LAMB SCORES BY EMPLOYMENT STATUS).

	(1)	(2)	(3)	(4)	(5)	(6)
	LaMB Factor 1: Collective purpose	LaMB Factor 2: Social contact	LaMB Factor 3: Status	LaMB Factor 4: Activity	LaMB Factor 5: Time structure	LaMB Factor 6: Financial strain
**Employment Status**						
Unemployed	Ref	Ref	Ref	Ref	Ref	Ref
Inactive	0.209***	0.250***	0.138***	0.425***	0.083**	-0.536***
	(0.031)	(0.027)	(0.031)	(0.029)	(0.027)	(0.026)
Minijob	0.387***	0.424***	0.252***	0.569***	0.128***	-0.519***
	(0.037)	(0.033)	(0.036)	(0.034)	(0.032)	(0.035)
Part time	0.470***	0.414***	0.221***	0.664***	0.147***	-0.809***
	(0.032)	(0.029)	(0.032)	(0.029)	(0.029)	(0.029)
Full time	0.451***	0.390***	0.272***	0.790***	0.134***	-1.032***
	(0.029)	(0.025)	(0.029)	(0.026)	(0.025)	(0.025)
Observations	9303	9303	9303	9303	9303	9303

Conditional on: age, age squared, gender, education, partner in HH, child (under 15) in HH.

* $p < 0.05$, ** $p < 0.01$, *** $p < 0.001$

TABLE A7 | REGRESSION RESULTS CORRESPONDING TO FIGURE 2 (Conditional on working: predicted scores by categories for hours worked) AND FIGURE 3 (Conditional on working: predicted scores by hourly wage categories).

	(1) LaMB Factor 1: Collective purpose	(2) LaMB Factor 2: Social contact	(3) LaMB Factor 3: Status	(4) LaMB Factor 4: Activity	(5) LaMB Factor 5: Time structure	(6) LaMB Factor 6: Financial strain
**Working time (hours per week)**						
<= 20	Ref	Ref	Ref	Ref	Ref	Ref
21-30	0.035	-0.005	0.123*	0.081	-0.082	-0.078
	(0.052)	(0.049)	(0.053)	(0.045)	(0.047)	(0.052)
31-35	0.069	0.071	0.188**	0.164***	-0.013	-0.148*
	(0.059)	(0.055)	(0.060)	(0.048)	(0.052)	(0.058)
36-40	0.072	0.006	0.162**	0.098*	-0.081	-0.246***
	(0.050)	(0.046)	(0.050)	(0.043)	(0.044)	(0.050)
41-45	0.049	-0.002	0.196***	0.199***	-0.130**	-0.218***
	(0.055)	(0.052)	(0.053)	(0.046)	(0.048)	(0.054)
>45	0.066	0.032	0.130*	0.296***	-0.217***	-0.350***
	(0.066)	(0.060)	(0.064)	(0.052)	(0.059)	(0.061)
**Hourly pay (Euros)**						
<= 10	Ref	Ref	Ref	Ref	Ref	Ref
11-15	0.031	0.034	0.071	0.001	0.036	-0.121**
	(0.047)	(0.041)	(0.046)	(0.038)	(0.042)	(0.045)
16-20	0.078	0.079	0.046	0.016	0.046	-0.389***
	(0.050)	(0.046)	(0.050)	(0.040)	(0.046)	(0.049)
21-25	0.140*	0.156**	0.065	0.022	0.002	-0.548***
	(0.056)	(0.053)	(0.059)	(0.045)	(0.052)	(0.054)
26-30	0.075	0.134*	0.058	0.077	0.037	-0.611***
	(0.067)	(0.062)	(0.064)	(0.050)	(0.061)	(0.064)
31-35	0.038	0.088	-0.017	-0.085	-0.166*	-0.640***
	(0.081)	(0.076)	(0.084)	(0.072)	(0.080)	(0.077)
36-40	0.124	0.263**	0.046	0.049	0.023	-0.827***
	(0.096)	(0.098)	(0.091)	(0.079)	(0.095)	(0.084)
>= 41	0.104	0.158*	0.103	0.068	0.009	-0.668***
	(0.081)	(0.075)	(0.073)	(0.067)	(0.077)	(0.078)
Observations	3442	3442	3442	3442	3442	3442

Conditional on: age, age squared, gender, education, partner in HH, child (under 15) in HH. Regular employees only.

* $p < 0.05$, ** $p < 0.01$, *** $p < 0.001$

TABLE A8 | REGRESSION RESULTS CORRESPONDING TO FIGURE 4 (CONDITIONAL ON UNEMPLOYMENT: PREDICTED SCORES BY MONTHLY UNEMPLOYMENT DURATION CATEGORIES).

	(1) LaMB Factor 1: Collective purpose	(2) LaMB Factor 2: Social contact	(3) LaMB Factor 3: Status	(4) LaMB Factor 4: Activity	(5) LaMB Factor 5: Time structure	(6) LaMB Factor 6: Financial strain
**Unemployment duration (months)**						
0-6	Ref	Ref	Ref	Ref	Ref	Ref
7-12	-0.114	-0.214*	-0.188	-0.185*	-0.047	0.302***
	(0.101)	(0.091)	(0.101)	(0.094)	(0.085)	(0.079)
13-18	-0.121	-0.148	-0.144	-0.057	-0.174*	0.397***
	(0.102)	(0.090)	(0.096)	(0.098)	(0.087)	(0.082)
19-24	-0.157	-0.183	-0.228*	-0.092	-0.185	0.397***
	(0.112)	(0.097)	(0.110)	(0.105)	(0.097)	(0.087)
25-30	-0.075	-0.195*	-0.160	-0.136	-0.053	0.375***
	(0.102)	(0.092)	(0.100)	(0.095)	(0.081)	(0.082)
31-36	0.013	-0.167	-0.155	-0.168	-0.008	0.289***
	(0.104)	(0.094)	(0.104)	(0.093)	(0.089)	(0.082)
37-42	-0.151	-0.363***	-0.293**	-0.169	-0.072	0.382***
	(0.111)	(0.097)	(0.109)	(0.103)	(0.100)	(0.086)
43-48	-0.033	-0.091	-0.209	-0.286**	-0.076	0.189*
	(0.121)	(0.101)	(0.114)	(0.107)	(0.101)	(0.094)
>= 49	-0.181*	-0.199**	-0.287***	-0.141	-0.184*	0.297***
	(0.083)	(0.075)	(0.082)	(0.078)	(0.073)	(0.070)
Observations	1601	1601	1601	1601	1601	1601

Conditional on: age, age squared, gender, education, partner in HH, child (under 15) in HH. Regular employees only.

* $p < 0.05$, ** $p < 0.01$, *** $p < 0.001$

TABLE A9 | Regressions of various outcomes on a set of controls and latent functions.

	(1) Health satisfaction (0-10)	(2) Health satisfaction (0-10)	(3) Subjective general health (1-5)	(4) Subjective general health (1-5)	(5) Good general health (0/1)	(6) Good general health (0/1)	(7) Subjective mental health (1-5)	(8) Subjective mental health (1-5)	(9) Good mental health (0/1)	(10) Good mental health (0/1)
Unemployed	-1.101***	-0.273***	-0.373***	-0.056	-0.155***	-0.028	-0.527***	-0.165***	-0.162***	-0.050**
	(0.072)	(0.076)	(0.035)	(0.038)	(0.016)	(0.018)	(0.043)	(0.046)	(0.016)	(0.018)
Inactive	-0.669***	-0.278***	-0.219***	-0.070*	-0.080***	-0.020	-0.342***	-0.173***	-0.116***	-0.064***
	(0.062)	(0.058)	(0.032)	(0.031)	(0.016)	(0.016)	(0.039)	(0.038)	(0.015)	(0.015)
Minijob	-0.170*	0.095	-0.081*	0.020	-0.055**	-0.014	-0.146**	-0.033	-0.063**	-0.029
	(0.078)	(0.075)	(0.040)	(0.039)	(0.020)	(0.020)	(0.049)	(0.049)	(0.020)	(0.020)
Part time	-0.141*	-0.046	-0.077*	-0.042	-0.032	-0.017	-0.133**	-0.093*	-0.051**	-0.039*
	(0.064)	(0.062)	(0.036)	(0.035)	(0.018)	(0.018)	(0.043)	(0.042)	(0.018)	(0.017)
Full time	Ref.	Ref.	Ref.	Ref.	Ref.	Ref.	Ref.	Ref.		
LAMB Factor 1: Collective purpose		0.366***		0.146***		0.052***		0.126***		0.038***
		(0.033)		(0.016)		(0.008)		(0.020)		(0.008)
LAMB Factor 2: Social contact		0.226***		0.086***		0.024**		0.120***		0.036***
		(0.035)		(0.017)		(0.008)		(0.022)		(0.008)
LAMB Factor 3: Status		-0.043		-0.033*		-0.004		-0.033		-0.006
		(0.034)		(0.016)		(0.007)		(0.021)		(0.008)
LAMB Factor 4: Activity		0.163***		0.060***		0.030***		0.084***		0.026***
		(0.030)		(0.014)		(0.006)		(0.018)		(0.007)
LAMB Factor 5: Time structure		0.227***		0.129***		0.048***		0.160***		0.056***
		(0.035)		(0.017)		(0.008)		(0.021)		(0.008)
LAMB Factor 6: Financial strain		-0.410***		-0.155***		-0.062***		-0.173***		-0.052***
		(0.030)		(0.015)		(0.007)		(0.018)		(0.007)
Mean: Full-time employed	7.375	7.375	3.536	3.536	0.556	0.556	3.906	3.906	0.689	0.689
Observations	9303	9303	9303	9303	9303	9303	9303	9303	9303	9303
R2	0.144	0.251	0.095	0.170	0.072	0.119	0.061	0.125	0.047	0.091
Indirect effect		-0.822		-0.316		-0.126		-0.362		-0.111
Share indirect (%)		75.465		84.943		81.662		68.296		68.607

Conditional on age, age squared, gender, education, partner in HH, child (under 15) in HH, interview mode.
$^{*}\ p < 0.05$, $^{**}\ p < 0.01$, $^{***}\ p < 0.001$

TABLE A10 | Average marginal effects (AMEs) from probit and logit regressions for binary outcomes corresponding to TABLE A9

	(1)	(2)	(3)	(4)	(5)	(6)	(7)	(8)
	Good general health (0/1)				Good mental health (0/1)			
	Logit	Logit	Probit	Probit	Logit	Logit	Probit	Probit
Reg Unemployed	-0.153***	-0.025	-0.154***	-0.027	-0.159***	-0.047**	-0.160***	-0.048**
	(0.016)	(0.018)	(0.016)	(0.018)	(0.016)	(0.017)	(0.016)	(0.017)
Inactive	-0.079***	-0.019	-0.080***	-0.021	-0.116***	-0.064***	-0.116***	-0.064***
	(0.016)	(0.015)	(0.016)	(0.015)	(0.015)	(0.015)	(0.015)	(0.015)
Minijob	-0.054**	-0.011	-0.054**	-0.013	-0.063***	-0.030	-0.063**	-0.031
	(0.020)	(0.020)	(0.020)	(0.020)	(0.019)	(0.019)	(0.019)	(0.019)
Part time	-0.031	-0.014	-0.031	-0.015	-0.051**	-0.040*	-0.051**	-0.039*
	(0.018)	(0.017)	(0.018)	(0.017)	(0.017)	(0.017)	(0.017)	(0.017)
Full time	Ref	Ref	Ref	Ref	Ref	Ref	Ref	Ref
LAMB Factor 1: Collective purpose		0.053***		0.053***		0.037***		0.038***
		(0.008)		(0.007)		(0.007)		(0.007)
LAMB Factor 2: Social contact		0.025**		0.025**		0.035***		0.035***
		(0.008)		(0.008)		(0.008)		(0.008)
LAMB Factor 3: Status		-0.004		-0.004		-0.005		-0.005
		(0.008)		(0.008)		(0.007)		(0.007)
LAMB Factor 4: Activity		0.032***		0.032***		0.026***		0.025***
		(0.007)		(0.007)		(0.007)		(0.007)
LAMB Factor 5: Time structure		0.052***		0.051***		0.055***		0.055***
		(0.008)		(0.008)		(0.008)		(0.008)
LAMB Factor 6: Financial strain		-0.061***		-0.061***		-0.050***		-0.050***
		(0.007)		(0.007)		(0.007)		(0.007)
Observations	9303	9303	9303	9303	9303	9303	9303	9303

Conditional on age, age squared, gender, education, partner in HH, child (under 15) in HH.

* $p < 0.05$, ** $p < 0.01$, *** $p < 0.001$

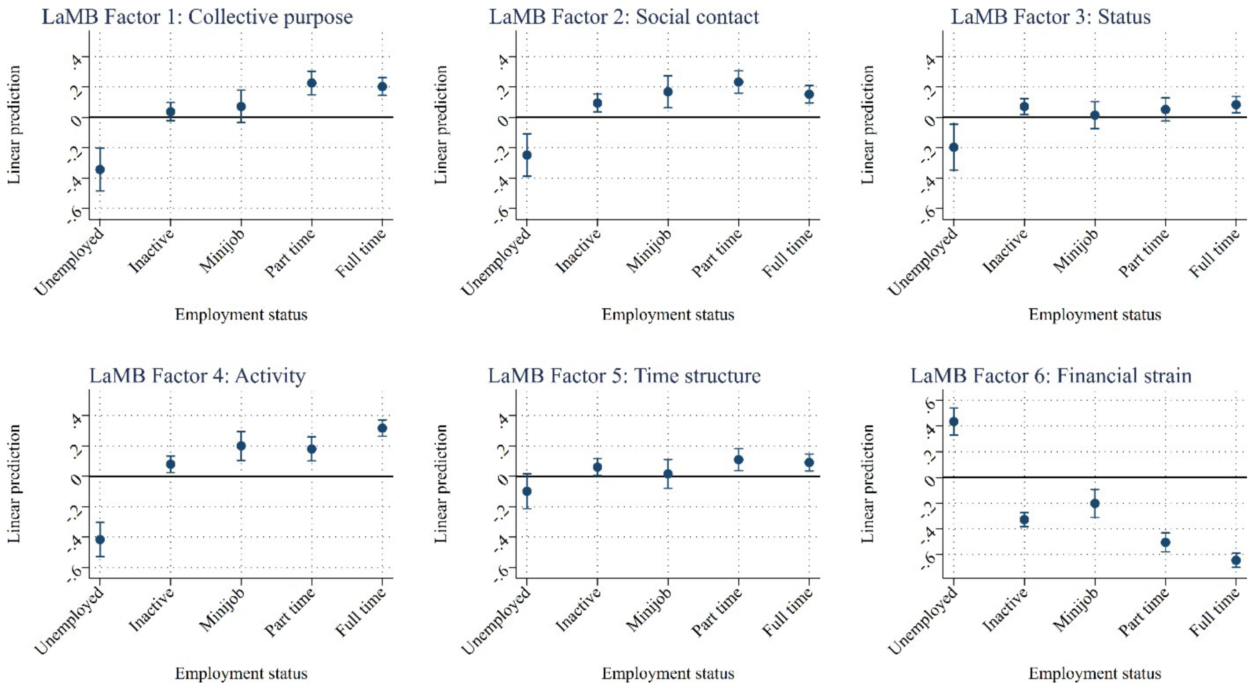

Figure A1 | Recalculation of Figure 1 (Standardized LAMB scores by employment status) using survey weights

Note: Means (SD)

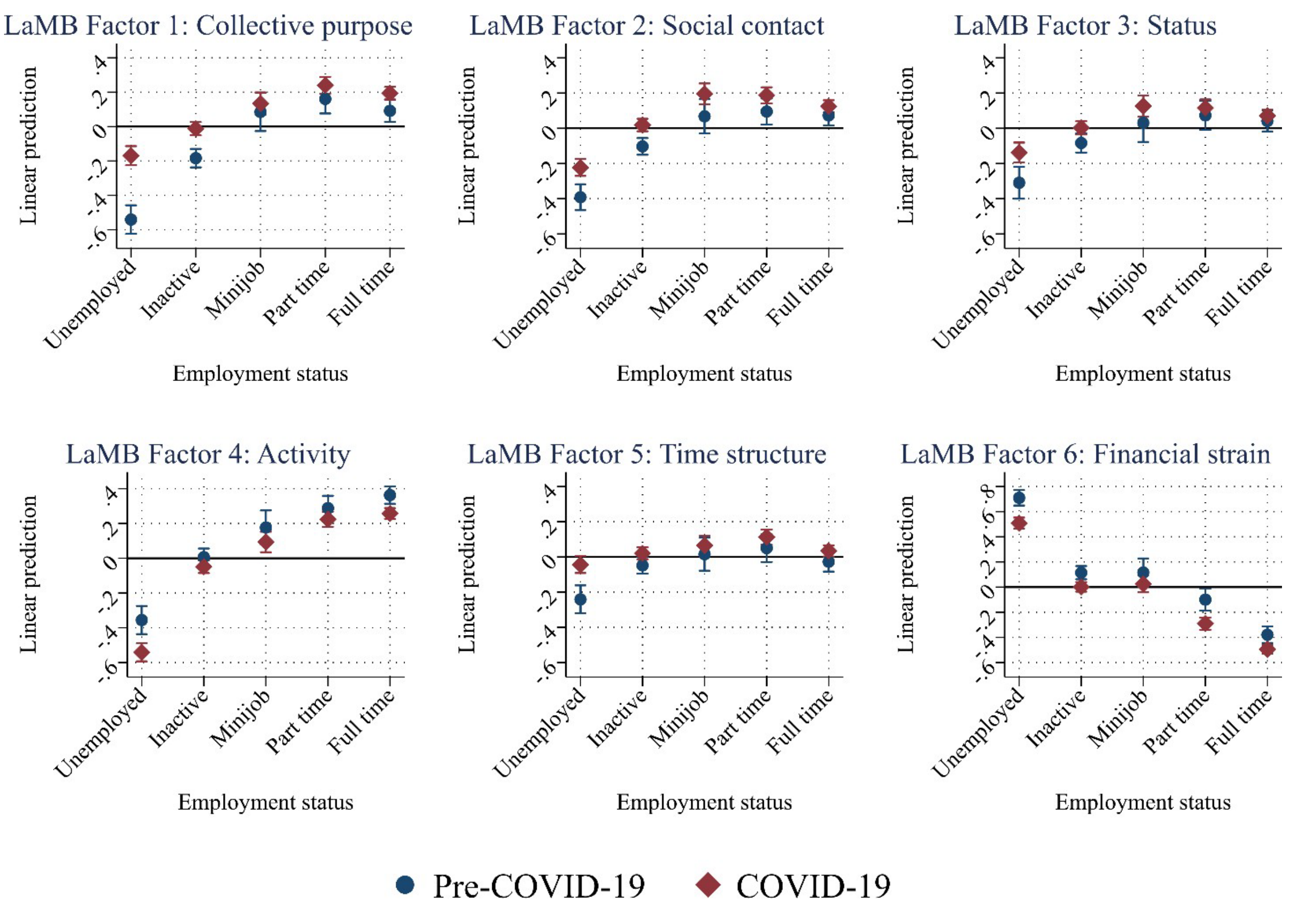

Figure A2 | Recalculation of Figure 1 (standardized LAMB scores by employment status), distinguishing between the periods before and after the COVID-19 containment measures

Note: Means (SD)

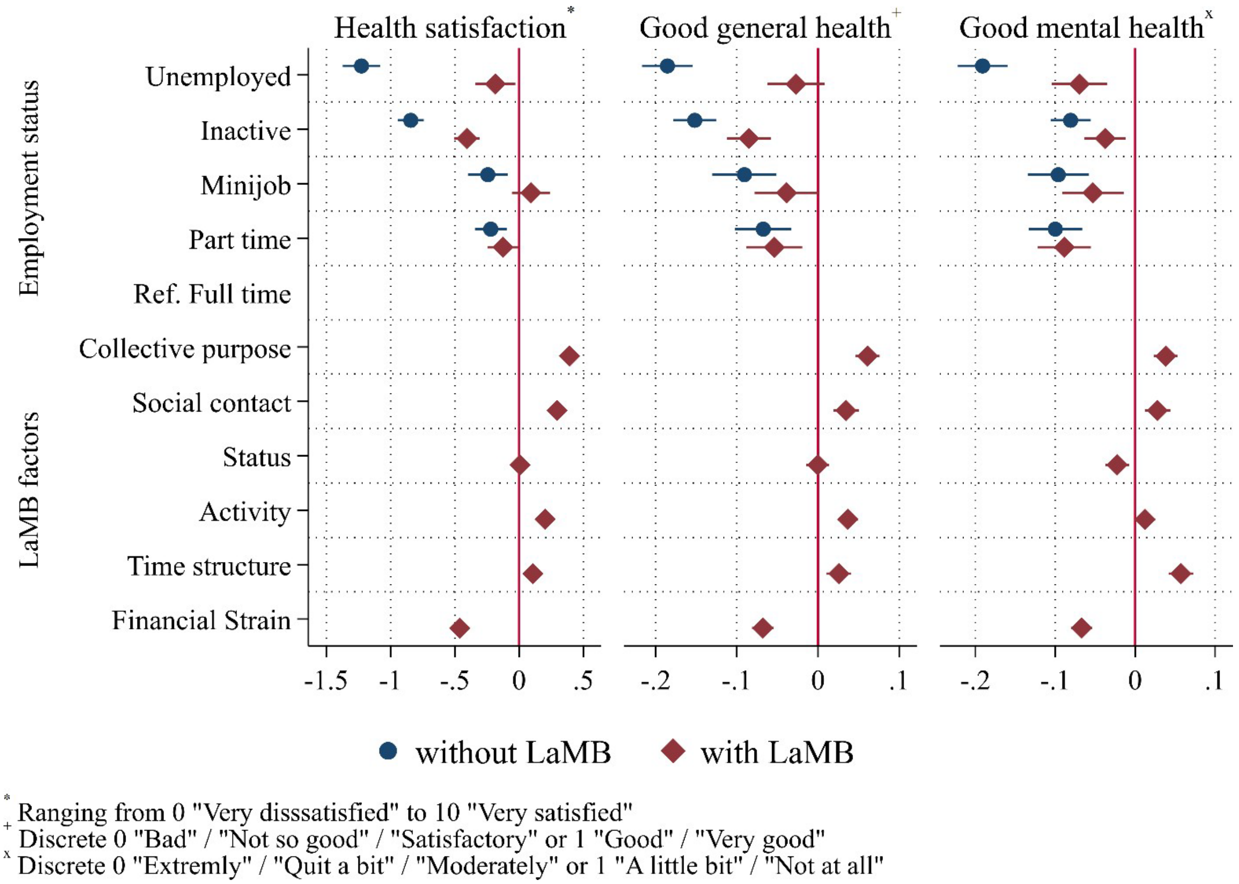

Figure A3 | Recalculation of Figure 5 (coefficient plot from regressions of well-being measures on employment status and LaMB factors) using binary indicators for the general and mental health measures

Note: 95% CI.

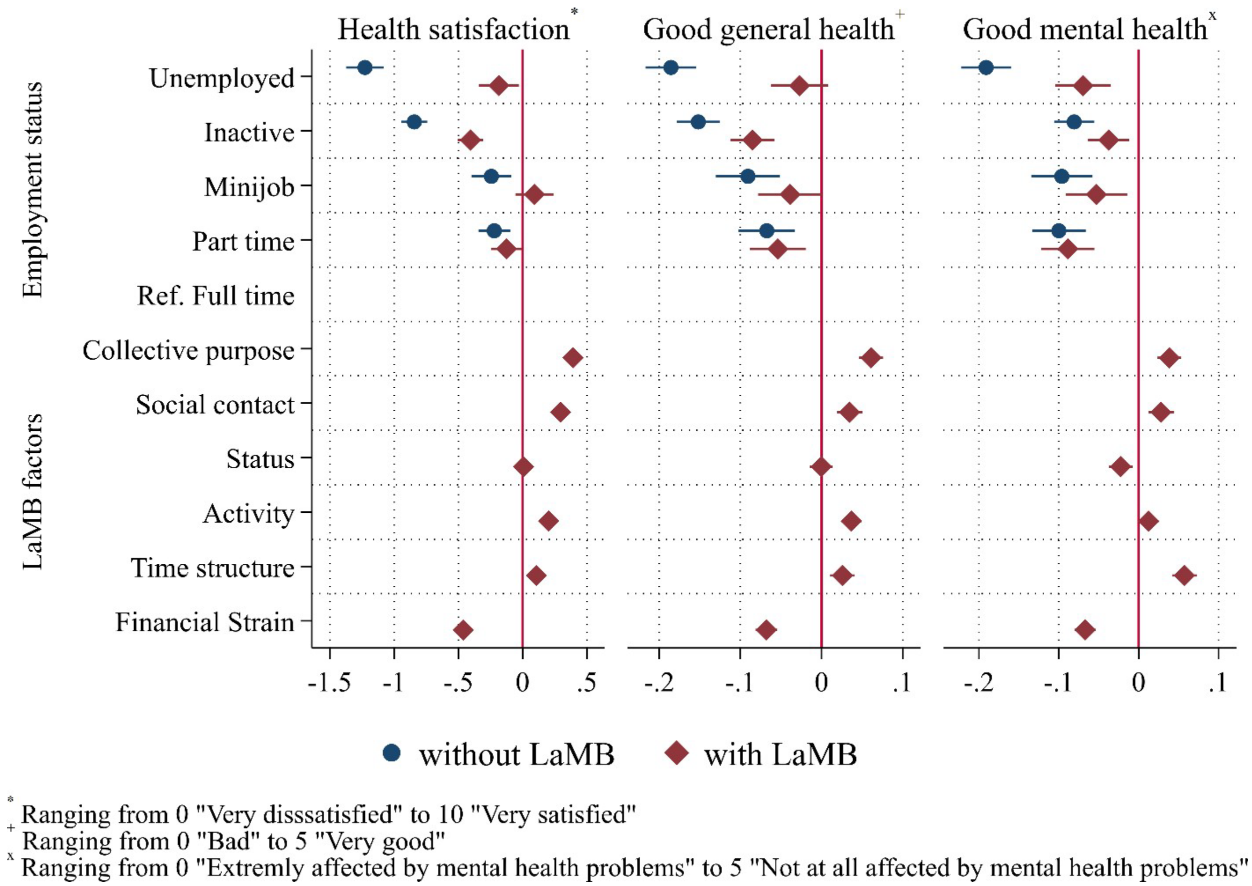

Figure A4 | Recalculation of Figure 5 (coefficient plot from regressions of well-being measures on employment status and LaMB factors) without any additional control variables

Note: 95% CI.
