# Supplementary material for: Heterogeneities in the latent functions of employment: New findings from a large-scale German survey
Source: Front Psychol. 2022 Aug 17;13:909558. doi: 10.3389/fpsyg.2022.909558 (PMC9428713; doi:10.3389/fpsyg.2022.909558)
Supplement: Supplementary file 2 [file Data_Sheet_2.zip › Readme_replication.docx]

**Syntax to “Heterogeneities in the latent functions of employment: New findings from a large-scale German survey”**

Sebastian Bähr, Bernad Batinic and Matthias Collischon

These files can be used to replicate the findings from our paper.

The following ado-files are required for the syntax to work:

- coefplot (create the Figures)
- esttab (display the results)
- fre (frequency tables)
- grc1leg (create the figures)
- oparallel (statistical tests)

We provide all ados at the versions used in this paper in the frontiers_LaMB_ados.zip file.

The PASS data can be obtained from the IAB Research Data centre:

<https://fdz.iab.de/en/FDZ_Individual_Data/PASS.aspx>

Stata 17 was used in the analysis (some of the commands used require Stata 17 to function properly).

The following Stata programs are included in this file:

- **frontiers_LaMB_01_data.do:**
  - prepares the PASS-SUF for the analysis regarding the LaMB-variables
  - Generates outcome and control variables
- **frontiers_LaMB_02_analysis.do:**
  - Creates all Tables and Figures in the article
